# Neuroanatomical organization of gonadotropin-releasing hormone neurons during the oestrus cycle in the ewe

**DOI:** 10.1186/1471-2202-5-46

**Published:** 2004-11-22

**Authors:** Martine Batailler, Alain Caraty, Benoît Malpaux, Yves Tillet

**Affiliations:** 1 Neurobiologie et Maîtrise des Fonctions Saisonnières, UMR 6175 INRA-CNRS-Université de Tours-Haras Nationaux, IFR 135, INRA Centre de Tours, 37380 Nouzilly, France; 2 Contrôle Central de l'Ovulation, UMR 6175 INRA-CNRS-Université de Tours-Haras Nationaux, IFR 135, INRA Centre de Tours, 37380 Nouzilly, France

## Abstract

**Background:**

During the preovulatory surge of gonadotropin-releasing hormone (GnRH), a very large amount of the peptide is released in the hypothalamo-hypophyseal portal blood for 24-36H00. To study whether this release is linked to a modification of the morphological organization of the GnRH-containing neurons, *i.e. *morphological plasticity, we conducted experiments in intact ewes at 4 different times of the oestrous cycle (before the expected LH surge, during the LH surge, and on day 8 and day 15 of the subsequent luteal phase). The cycle stage was verified by determination of progesterone and LH concentrations in the peripheral blood samples collected prior to euthanasia.

**Results:**

The distribution of GnRH-containing neurons throughout the preoptic area around the vascular organ of the lamina terminalis was studied following visualisation using immunohistochemistry. No difference was observed in the staining intensity for GnRH between the different groups. Clusters of GnRH-containing neurons (defined as 2 or more neurons being observed in close contact) were more numerous during the late follicular phase (43 ± 7) than during the luteal phase (25 ± 6), and the percentage of clusters was higher during the beginning of the follicular phase than during the luteal phase. There was no difference in the number of labelled neurons in each group.

**Conclusions:**

These results indicate that the morphological organization of the GnRH-containing neurons in ewes is modified during the follicular phase. This transitory re-organization may contribute to the putative synchronization of these neurons during the surge. The molecular signal inducing this plasticity has not yet been identified, but oestradiol might play an important role, since in sheep it is the only signal which initiates the GnRH preovulatory surge.

## Background

Gonadotropin-releasing hormone (GnRH), the peptide responsible for the regulation of secretory activity of the pituitary gonadotropes, is found in a diffuse neuronal system situated in the preoptic area and anterior hypothalamic area [1]. The neurons project their terminals to the median eminence where the peptide is released into the hypothalamo-hypophyseal portal vessels. The secretion of GnRH is normally pulsatile, as demonstrated by the measurement of GnRH following the direct sampling of hypothalamo-pituitary portal blood in various species such as sheep [2,3]. The frequency of this pulsatility is the main characteristic of this secretion and it encodes each part of the sexual cycle in the female [4]. The highest circulating concentration of GnRH is induced during the follicular phase in the female by the sequential action of the two main ovarian steroids (progesterone and oestradiol). These act within the brain to trigger a large and sustained period of GnRH release, the GnRH surge, which stimulates the preovulatory LH surge, and subsequently ovulation.

The pulsatile nature of GnRH secretion, as well as the large amount of peptide released during the preovulatory surge, indicate that the activity of GnRH-containing neurons can be synchronized, and it has been demonstrated that the GnRH neurons of the preoptic area receive synaptic contacts from GnRH neurons [5-7]. Oestradiol, which initiates the preovulatory surge of GnRH, has been shown to be involved in the synaptic plasticity demonstrated in various neuronal populations, including those in the arcuate nucleus [8,9]. Moreover, in rats, an increase in the expression of the molecular markers of synaptic remodelling is observed at the time of the surge in the preoptic area where most of the GnRH neuron cell bodies are found [10]. In sheep, changes in the expression of the polysialylated form of the neural cell adhesion molecule around the periphery of the GnRH cell bodies, associated with the seasonal changes in GnRH secretion, are indicative of potential plastic changes in this neural system linked with changes in secretory activity [11]. In addition, oestradiol and progesterone have been shown to modify the morphology and staining intensity of GnRH neurons within the ovine hypothalamus [12], suggesting that ovarian steroids could elicit plastic changes in GnRH neurons during the oestrous cycle. However, very little work has been done regarding neuronal organization at the level of the GnRH cell bodies during the oestrous cycle in the ewe.

The aim of this work was to study the number, distribution and potential contacts present between GnRH-containing cells in intact ewes under natural conditions, focusing particularly on the neuroanatomical organization of these neurons around the preovulatory surge when a high level of oestradiol is released by the growing follicles.

## Results

### Plasma hormones

**Figure 1:**
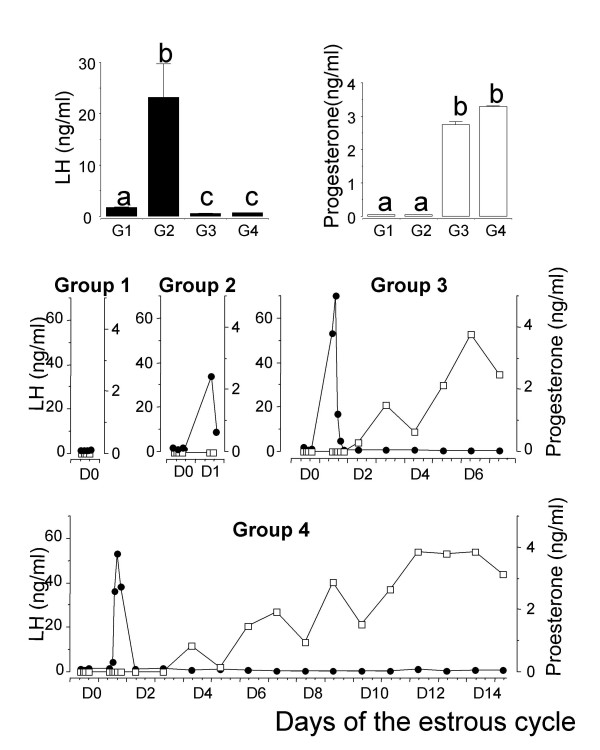
Top: Plasmatic hormone concentrations (Mean ± SEM) for LH (black bars) or progesterone (open bar) at the last sampling time before animals were sacrificed. Different letters indicate differences between groups (P < 0.05). Bottom: Individual plasmatic levels of LH (black circles) and progesterone (open squares) in ewes representative of each experimental group.

Because there are some individual variations in the response to the induction of ovulation treatment (oestrus onset, preovulatory LH surge onset, etc.), an analysis of plasma hormone concentration was carried out to confirm the allocation of animals to experimental groups according to the characteristic hormonal level previously described [13]. In group 1, as illustrated in figure 1, progesterone and LH concentrations were low, characteristic of the late follicular phase. In group 2, progesterone remained low (less than 0.05 ng/ml) and a surge of LH was observed for 4 out 5 animals. Analysis of the LH profile concentrations showed that these animals were killed either during the ascending (n = 2) or descending (n = 2) part of the LH surge; for the remaining animal only a slight increase in LH secretion was observed at the end of the sampling period. In groups 3 and 4 a preovulatory surge of LH was clearly identified in all animals. These LH surges were followed by a clear increase in progesterone concentrations and the progesterone level was in the range of 2.5 to 4.5 at the end of the sampling period (except for one animal 1.9 ng/ml in group 4). Global analysis of plasma hormone concentrations at the last sampling time before animals were killed revealed significant differences between groups for LH (p < 0.01) and progesterone (p < 0.05). As shown in figure 1, the LH level was higher in group 2 than for any other group (p < 0.01), and progesterone concentrations were lower in groups 1 and 2 than in groups 3 and 4 (p < 0.01). For groups 1, 2, 3 and 4, mean levels ± SEM were respectively 1.07 ± 0.12, 28.20 ± 7.08, 0.56 ± 0.03, 0.65 ± 0.06 for LH and 0.05 ± 0.0, 0.05 ± 0.0, 2.74 ± 0.10, 3.62 ± 0.26 for progesterone. However, the ewes showing no clear LH surge (group 2) or a moderate increase in progesterone concentration (group 4) were considered as slightly different from any other animal of the same group and were not used for determining the number or relative clustering of GnRH neurons.

Four of the eight animals in experiment 2 were killed during the LH surge (2 during the ascending and 2 during the descending part). The 4 remaining animals were killed 4–6 hours after the end of the LH surge and were discarded from the study.

### GnRH-immunoreactive neurons

#### Experiment 1

**Figure 2:**
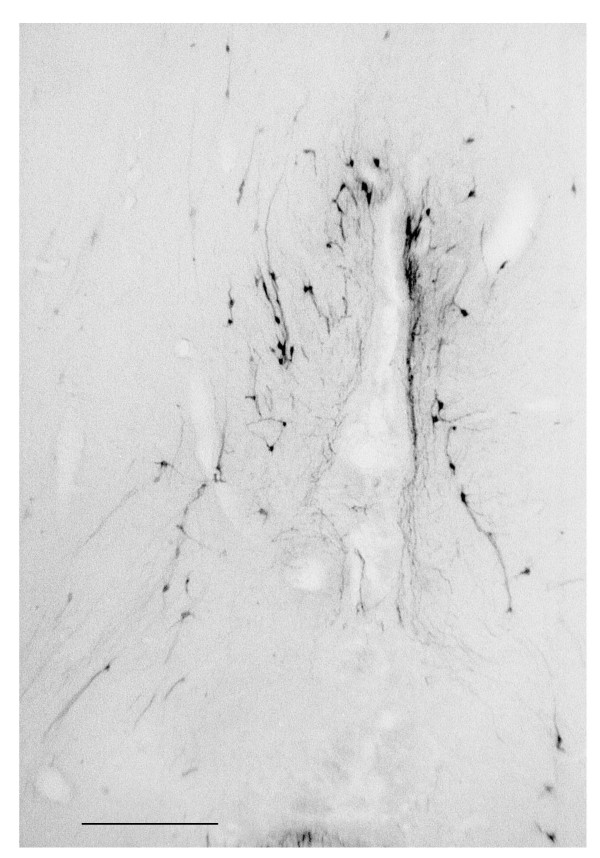
GnRH-immunoreactive neurons in the preoptic area where the highest number of GnRH-immunoreactive neurons was found. Scale bar: 400 μm

GnRH-containing neurons were distributed in the preoptic area (fig 2) as previously described [14]. There was some variation in the staining intensity in the same section, but it was usually strong. The black DAB/Nickel ammonium sulfate precipitate was distributed throughout the cytoplasm in the cell bodies and processes. The nucleus was never stained. GnRH-ir fibres were also observed around perikarya, presenting large varicosities. The distribution of GnRH cell bodies was homogenous between animals. GnRH-ir neurons were counted when the nucleus was clearly visible.

**Figure 3:**
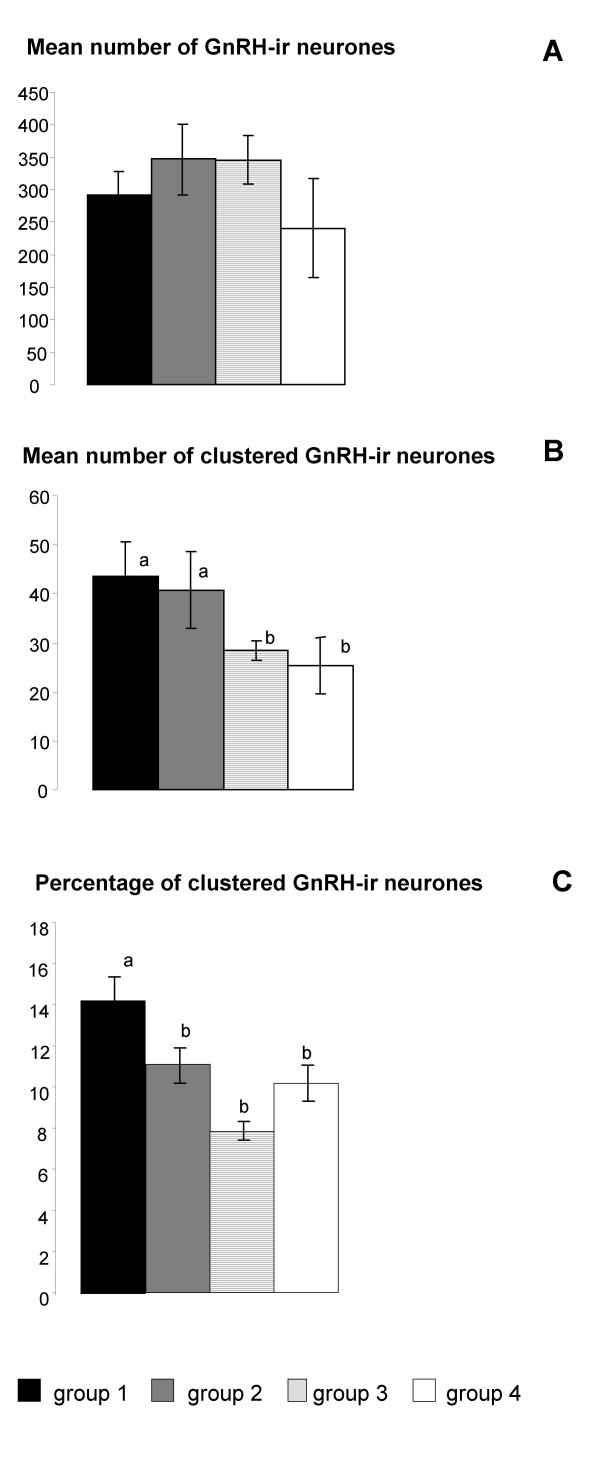
Mean number (±SEM) of single (A), clustered (B) and percentage of clusters (C) of GnRH-immunoreactive neurons counted in the preoptic area of ewes in the four experimental groups of the first experiment. Different letters indicate significant differences (p < 0.05). On histogram B, groups 1 and 2, like groups 3 and 4, are not statistically different, but groups 1 and 2, and groups 3 and 4, are statistically different; on histogram C group 1 is statistically higher than the other groups.

**Figure 4:**
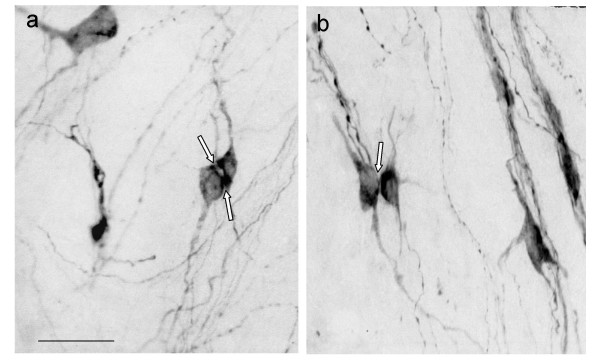
GnRH-immunoreactive neurons in the preoptic area of ewes. **a **and **b **represent two examples of clusters of GnRH-immunoractive neurons, white arrows indicate putative contacts between the neurons. Scale bar: a, b – 40 μm

There was no difference in the total number of GnRH-ir neurons in the experimental groups (group 1: 291 ± 38, n = 5, group 2: 347 ± 41 n = 4, group 3: 345 ± 38 n = 5, group 4: 241 ± 75 n = 4) (fig 3A). However, the organization of the labelled neurons differed in the different experimental groups. Clusters of two or more GnRH-ir neurons in which there appeared to be close contacts between neuronal cell bodies (figs 4a,4b) were found in the preoptic area of all groups. However, the number of clusters and the proportion of identified neurons involved in clusters varied significantly between the experimental groups. These clusters (mainly pairs of neurons) were more numerous in groups 1 (43 ± 7) and 2 (41 ± 8) at the moment of the preovulatory surge than in groups 3 (28 ± 2) and 4 (25 ± 6) in the luteal phase (p < 0.05) (fig 3B).

The percentage of clusters was higher in group 1 (15.1 ± 1.2) than in groups 2 (11.8 ± 1.0), 3 (8.3 ± 0.4) and 4 (10.7 ± 0.9) (fig 3C).

#### Experiment 2

In the second experiment, four additional animals were studied during the GnRH surge, and the data compared with those of the group 2 ewes in the first experiment. As shown in table 1, there was no difference in the proportion of clusters of GnRH-ir neurons in the four animals sampled during the ascending phase of GnRH surge and the four sampled during the descending phase of the surge.

## Discussion

Our observations indicate that the organization of GnRH-ir neurons was modified during the oestrous cycle, since more clusters were observed during the follicular phase. However, we did not observe any difference between the ascending and descending phases of LH release in group 2. Therefore, our data support the hypothesis that there are changes in communication between GnRH cell bodies prior to the initiation of the preovulatory GnRH surge.

The increase in the number of clusters was not mirrored by a parallel change in the total number of GnRH cell bodies. However, as the number of clusters was relatively small compared to the GnRH cell population (constituting 10% to 15%), the putative increase in the number of GnRH-ir neurons could have been masked due to the large variations in the number of labelled neurons between animals. An alternative hypothesis is that the number of GnRH neurons remains constant, and that the variations in the number of clusters is linked to variations in the peptide level, which are consistent with the variations in the staining intensity observed in all animals. In this case, the level of GnRH would be higher in clustered neurons than in isolated neurons at the moment of the preovulatory surge, and therefore neuronal communication would be more efficient in clustered neurons. The percentage of clusters increased only in group 1, while the difference between the number of clusters in groups 2, 3 and 4 was not statistically significant. This special morphological organization may constitute an anatomical support for the synchronization of GnRH neuronal activity needed to induce the GnRH preovulatory surge.

Clusters of GnRH-ir cell bodies have been previously described in sheep [15] (Lehman et al, 1986), rats and monkeys [16]. In rats they represented only 2–7% of the GnRH neuronal population [16]. In our study, the proportion of clusters was similar (10–15%) to that observed in monkeys (3–15%). However, contrary to our observation, the numbers did not change with the different hormonal conditions in the monkey [16]. This might be related to the distribution of GnRH cell bodies which differs between monkeys and sheep [17], and it is possible that the increase of clusters observed in our experiments was related to a specific GnRH neuronal population involved in the GnRH surge secretion. Indeed, Fos expression at the time of the preovulatory LH surge has been shown to be expressed in a subset of GnRH neurons in rats [18].

A neuronal re-organization of GnRH-containing neurons has also been previously described in anoestrous ewes where the neurons are ensheathed by glial processes which decrease the number of axo-somatic synaptic contacts [19]. In this latter situation, photoperiod would be the major signal to induce such a modification. Another example of the relationship between synaptic afferents and GnRH secretion has been described in the monkey where the increase of GnRH activity at puberty correlates with the decrease in synaptic afferents on GnRH-containing neurons [20]. This morphological regulation of GnRH-containing neurons, through synaptic contacts or perikaryal apposition, involves glial processes, since in both sheep and monkeys these neurons are ensheathed by numerous glial processes [19,21].

Steroids, mainly oestradiol, are the most potent regulators in the control of GnRH neuronal activity. Therefore, we may hypothesize that this "plasticity" results from an oestradiol effect, as has been shown in the monkey where oestradiol modulates the number of synaptic contacts on GnRH neurons [22]. In rats and monkeys, numerous studies have demonstrated that oestrogens induce synaptic plasticity in the control of gonadotropin secretion (rat: [23]; monkeys: [24]). In our study, the highest number of clusters was found during the preovulatory surge which occurred about 24 hours after the expected oestradiol increase, this delay being consistent with an oestradiol effect.

There was no difference in the number of labelled neurons in the experimental groups, and this observation may indicate that the level of GnRH-immunoreactivity was stable in the neurons of the preoptic area. It has previously been demonstrated in the ewe that GnRH messenger ribonucleic acid expression changes before the onset of the oestradiol-induced luteinizing hormone surge [25], and that the staining intensity of GnRH-ir perikarya increases after oestradiol treatment, but that the number of labelled neurons does not change [12,26]. In addition, the variations of LH secretion induced by progesterone withdrawal are not linked to a variation in GnRH mRNA [27]. Unlike the apparent stability of the GnRH level in the perikarya, GnRH-immunoreactivity decreases in the median eminence after ovulation [28]. This information could indicate that variations in GnRH secretion arise from its release from the median eminence terminals without variations in peptides in the perikarya.

## Conclusions

In conclusion, we have demonstrated that the morphological organization of the GnRH neurons of the preoptic area is modified during the oestrous cycle, although the overall number of GnRH-ir neurons does not change. During the preovulatory surge, the GnRH neurons are more frequently found in clusters, and the percentage of clusters is significantly higher immediately prior to the preovulatory LH surge. Actual contacts between GnRH-ir neurons cannot be determined using light microscopy, but can only be demonstrated using electron microscopy. Because oestradiol is the most powerful regulator of GnRH activity during the oestrous cycle, we can hypothesize that this plasticity may be induced by steroids. The role of this plasticity remains to be demonstrated, but it could increase interneuronal communication during the preovulatory surge.

## Methods

All experimental procedures were carried out in accordance with authorisation A37801 of the French Ministry of Agriculture.

### Animals

Intact adult Ile de France ewes (n = 28) from the laboratory flock, maintained under natural photoperiod were used. Two experiments were performed (one year apart) during the breeding season (between November 15^th ^and December 15^th^). Animals were fed daily with hay, straw and corn, and water was available *ad libitum*. All of the ewes had lambed at least once.

### Experimental design

#### Experiment N° 1

Animals (n = 20) were killed at specific time points in the oestrous cycle. In order to synchronize oestrous cycles, animals were treated with an intravaginal progesterone-releasing device (CIDR InterAg, Hamilton, New Zealand) for 14 days. Following removal of the progesterone device, animals were treated (i.m.) with 500 UI of Pregnant Mare Serum Gonadotropin (PMSG), a treatment known to induce the LH surge and ovulation in sheep (for review see [29]). It has been shown that oestrous behaviour in this breed precedes the LH surge by 7.0 ± 1.6 hours [30]. Therefore, oestrous behaviour was recorded for each ewe every 6 hours, from 18 hours after PMSG administration until the animal was observed to be in oestrus using a ram wearing an apron. The animals were then allocated to four groups:

Group 1: Animals killed 1–2 hours after oestrus, i.e. before the LH surge onset, (n = 5)

Group 2: Animals killed 8–12 hours after oestrus, i.e. during the preovulatory LH surge, (n = 5)

Group 3: Animals killed 8 days after the preovulatory LH surge, (n = 5)

Group 4: Animals killed 15 days after the preovulatory LH surge, (n = 5)

#### Experiment N° 2

Analysis of LH secretion (see paragraph below) revealed that animals in group 2 in experiment 1 were killed either in the ascending (n = 2) or descending (n = 2) phase of the LH surge. Therefore, in order to increase the N for each sub-group, 8 animals were synchronised as previously described (one year later, during the breeding season) and killed 12 and 20 hours (n = 4) after oestrus.

For both experiments, blood samples were collected by venepuncture every 2 hours from 18 hours after PMSG administration until sacrifice (groups 1 and 2) and daily thereafter (groups 3 and 4).

### Hormone assays

Plasma samples were assayed in duplicate for LH with 100 μl aliquots of plasma using a previously described RIA method [31]. All samples from one experiment were run in a single assay. Intra-assay coefficient of variation averaged 9% and assay sensitivity was 0.16 ± 0.05 ng/ml (4 assays) of standard 1051-CY-LH (i.e. 0.31 ng/ml of NIH-LH-S1). Progesterone was determined in a single assay after hexane extraction of 100 μl of plasma [32]. The sensitivity was 5 pg/tube and the intra-assay CV 10%.

### Immunohistochemistry

A solution of heparin (25,000 units) was injected *iv *10 min before decapitation. Both carotids were catheterised and the head was perfused with 2 litres of 1% sodium nitrite followed by 4 litres of 4% paraformaldehyde in 0.1 M phosphate buffer (pH 7.4). After perfusion, the brain was quickly removed and a block containing the whole preoptic area was isolated and post-fixed for 2 hours at +4°C in the same fixative. The blocks were immersed in 30% sucrose containing 0.1% sodium azide at +4°C for 5 days.

Serial 40 μm coronal sections were obtained using a freezing microtome and stored at +4°C in PBS containing 0.1% sodium azide. Every fifth section was stained for GnRH immunoreactivity.

Sections were incubated for 2 hours at +4°C in PBS containing 0.3% triton X100 and 1% H_2_O_2 _and then preincubated for 30 min at room temperature in blocking solution (PBS, 6% normal sheep serum, 0.3% triton, 0.1% sodium azide). Sections were exposed to GnRH primary antiserum at a dilution of 1/10,000 in PBS containing 0.3% triton, 0.2% HSA, 0.1% sodium azide for 4 days at +4°C. After washing in PBS, sections were incubated for 3 hours at room temperature in the secondary antibody (1/500 with PBS containing 0.2% BSA). After washing in PBS, sections were incubated in rabbit peroxidase-antiperoxidase complex diluted 1/10,000 in PBS containing 0.2% BSA overnight at +4°C. After washing twice in PBS and in Tris buffer (0.05 M pH 7.6), the sections were reacted with 3,3-diaminobenzidine tetrahydrochloride (0.02%) and nickel ammonium sulfate (0.25%) in the same Tris buffer containing 0.0025% H_2_O_2 _for 20 min. The reactions were stopped by rinsing the sections with Tris buffer. Sections were mounted onto gelatine-coated slides, dried, counterstained with a solution of neutral red for 5 min and mounted with a cover-slide using Depex.

The characteristics and the specificity of the GnRH antibody were as previously described [14].

### Data analysis

The LH surge was assumed to start when LH concentration exceeded 6 ng/ml of plasma, *i.e. *about twice the maximum value of a pulse during the follicular phase for this breed and for this LH assay. For hormone values, a global comparison was carried out using a non-parametric ANOVA with exact general score test, and comparison between groups was carried out using the non-parametric exact permutation test for independent samples (Stat Exact Software, Cytel Software Corporation, Cambridge, MA, USA).

The first observation of the sections allowed the immunoreactive area surrounding the OVLT to be determined (fig. 2). We selected two sections where the size of the OVLT was maximum in the third ventricle, and the two rostral and caudal sections were studied. A total of six sections at 160 μm intervals were studied for each ewe. In each section, all immunoreactive neurons for GnRH were counted using a light microscope, and groups of closely-associated neurons were noted. Total number of cells and number of clusters were analyzed by a 2-way (group and section number) ANOVA (SuperANOVA, Abacus concept, California) followed by a student-Newman-keuls post-hoc test for two by two comparisons. The percentage of cells forming clusters was calculated for each animal. It was analyzed by a one-way (group) ANOVA after arcsin transformation followed by a student-Newman-keuls post-hoc test for two by two comparisons. The results are expressed as means ± standard error of the means.

## Authors' contribution

MB performed the immunohistochemical reaction and counted the neurones, AC and YT conceived the study, and participated in its design, coordination and in the collection of biological samples. BM participated in the final statistical analysis of results and corrected the manuscript.

## Figures and Tables

**Table 1:** Mean number of isolated or clustered GnRH neurons counted during and after the preovulatory LH surge. Individual values are indicated for each animal during the first (Exp I) and second experiment (Exp II).

	**Ascending phase of the surge**				**Descending phase of the surge**			
	
	**Exp I**		**Exp II**		**Exp I**		**Exp II**	
**Ewe identification n°**	**376**	**23**	**18**	**61**	**280**	**106**	**47**	**59**
Single GnRH neurons	323	306	383	352	331	427	354	463
**Clusters of GnRH neurons**	34	47	79	85	34	48	67	103
**%**	*10*	*15*	*20*	*23*	*10*	*11*	*19*	*22*
